# Screening of novel peptides that specifically interact with vitamin D bound biocomplex proteins

**DOI:** 10.1038/s41598-023-28881-w

**Published:** 2023-02-06

**Authors:** Taehwan Kim, Jaewoong Lee, Jin-Pyo Lee, Bit-Na Kim, Yang-Hoon Kim, Youn-Sik Lee, Jiho Min

**Affiliations:** 1 Graduate School of Semiconductor and Chemical Engineering, Jeonbuk National University, 567 Baekje-daero, Deokjin-gu, Jeonju-si, Jeollabuk-do 54896 Republic of Korea; 2 School of Biological Science, Chungbuk National University, Chungdae-ro 1, Seowon-gu, Cheongju, Chungbuk 28644 Republic of Korea; 3 School of Chemical Engineering, Jeonbuk National University, 567 Baekje-daero, Deokjin-gu, Jeonju-si, Jeollabuk-do 54896 Republic of Korea

**Keywords:** Biological techniques, Biotechnology

## Abstract

The majority of the vitamin D that is present in the blood binds to vitamin D binding protein (VDBP) and circulates in the form of a complex (VDBP-Complex). Knowing the level of vitamin D in the body is crucial for vitamin D-related treatments so that the right dosage of vitamin D can be given. In other words, it is essential to distinguish between the protein VDBP and the complex form bound to vitamin D. As a novel way for the detection of VDBP-Complex, a more effective phage display methodology was applied in this study along with the addition of two approaches. In order to screen a sequence specific to the target only, the pre-binding method and after-binding method were performed. VDBP-Complex was directly coated on the petri dishes. In order to select phages that specifically bind to the VDBP-Complex, random phages were attached, and selected by 7 times of biopanning. Individual DNA sequences were analyzed for each biopanning to find specific peptide sequences for VDBP-Complex. The affinity of binding phages was verified by ELISA assay using an anti-M13 antibody. The phage having a sequence of SFTKTSTFTWRD (called as M3) has shown the highest binding affinity to VDBP-Complex. As a result of the removal test of VDBP-Complex using magnetic beads conjugated with M3 peptide, it was confirmed that significant decrease of VDBP-Complex. The unique characteristic of the M3 sequence was confirmed through a sequence-modified peptide (SFT motif). That is, it is expected that the M3 peptide may be used to determine the vitamin D levels in the blood.

## Introduction

Vitamin D is an essential substance for the regulation of calcium and phosphorus homeostasis in humans. According to recent studies, it is associated with cell differentiation and cancer. In addition, it plays an important role in the regulation of the immune system, which is attracting attention due to coronavirus disease (COVID-19)^1,2^. About 80% of vitamin D is produced in the skin by a pathway that is synthesized when the skin is exposed to UVB radiation of sunlight. The remaining 20% of vitamin D is obtained by consuming food^3^. That vitamin D is converted to 25-hydroxyvitamin D in the liver, and subsequently converted to the 1,25-dihydroxyvitamin D as an active form with the vitamin D binding protein (VDBP) in the kidney^4^. However, modern people spend a lot of time indoors, so they are less exposed to sunlight, and do not even take vitamin D separately. For this reason, the concentration of vitamin D in the blood has gradually lowered in recent years, and vitamin D deficiency is an important problem in Koreans^5,6^. Vitamin D deficiency can cause mild pain, such as muscle aches, but can also cause serious bone disorders, such as rickets in children or osteomalacia in adults^7^. Vitamin D toxicity from excessive vitamin D overdose also exists. The clinical manifestations of vitamin D toxicity are a consequence of hypercalcaemia, and include fatigue, anorexia, vomiting, confusion, irritability, drowsiness, and coma^8^. It is vital to administer a suitable quantity that is not toxic for this reason.

For the measurement of vitamin D concentration, this vitamin D is difficult to detect. This is because vitamin D exists in the blood by forming a complex with the VDBP, rather than alone. To put it another way, vitamin D binding protein complex (VDBP-Complex) is a combination of VDBP and VD. Among them, in animal studies, VDBP not only acts as a transport protein, but also protects 25-hydroxyvitamin D from degradation, prolongs its half-life, and ultimately protects against vitamin D deficiency^9^. Due to the characteristic that vitamin D does not move alone in the blood, it has become important to detect the complex form for the detection of vitamin D^10^. Attempts were made to use antibodies to detect vitamin D. In detecting vitamin D, the antibody has more than 150 kDa of molecular weight, so it is observed that there is a problem in detecting small vitamin D. Also, in the case of antibodies specific to the VDBP-Complex, there is the problem that VDBP-Complex and VDBP cannot be distinguished^11^. Therefore, in this study, it was decided to characterize and identify sequences by screening phages that specifically bind to VDBP-Complex rather than directly targeting VD, to discover peptides that will be applied to checking the concentration of vitamin D in the blood. VDBP is a protein that is not as small as 55 kDa, so a small peptide was used to attach it. Thus, the phage display technique turned out to be suitable, as it uses a peptide of 8 kDa size. Originally, the phage display technique was generally used to identify peptide motifs with high and specific binding affinity for desired target materials, such as enzymes and proteins^12^. This technique was created by G. Smith in 1985 as a method for using numerous identical peptides expressed on the surface of filamentous bacteriophage^13^. The numerous identical peptides are expressed on the outside of the phage^14^. This allowed a physical bond between the peptide sequence and the DNA corresponding to it, allowing access to the target molecule through an in vitro selection process called panning^15^. Recently, applications of this technique have been extended widely to multiple non-biological implementations, such as nanobatteries^16^, nanorods^17^, or analytical biosensing^18^, as well as fields of biological research, such as drug discovery, diagnostics, biotechnology, and protein engineering^19–21^. To use this phage display technique, we utilized the widely used M13 phage library whose vectors are modified for the pentavalent display of peptides as N-terminal fusions to the minor coat protein pIII by a short linker GGGS^14^.

In this study, an empty petri dish was used as a negative control to carry out a phage display. VDBP-Complex and VDBP were used as vitamin-related biocomplexes, and they were diluted with NaHCO3 to coat targets using electrostatic attraction. The four steps of target coating, phage binding, phage elution, and phage amplifying are called biopanning. The biopanning cycle is the most important in phage display. For phage binding, the construction of a reliable phage library is required. The disadvantage of phage display is that effective antibodies can be identified only when a high diversity library is secured, in other words, it may take a long time to find a desired phage among numerous phages^22^. In addition, the traditional phage display simply proceeds with the phage elution after phage binding, which may cause unwanted non-specific binding phages. The phage display method to be used in this study was designed by adding two new experimental methods (the pre-binding method and the after-binding method) in the biopanning of the traditional phage display. To explain, the pre-binding method preferentially removes phages attached to the control group, and the after-binding method removes the phages that bind to the target and control group simultaneously. It was performed 7 times. Generally, phage display experiments were mainly performed in batch mode, resulting in only one equilibrium state hence low-efficiency selection. To overcome these drawbacks, there is a report monitoring the selection process using chromatographic biopanning with the use of a monolithic column^23^. However, in the chromatographic biopanning process of the report, there is a process of re-amplifying the phage after negative screening on an uncharged monolithic column, raising concerns about the amplification of non-specific binding phages that are not removed. In our study, there is no process of amplifying phages between pre-binding and biopanning to the target in one equilibrium state, so there is a possibility to more reliably remove non-specific binding phages. This newly designed phage display method can effectively screen phage sequences that are more selectively attached to their targets. In 7 times of revised biopanning, screening was performed in a random phage library to find a sequence that specifically binds to the VDBP-Complex. Then, the binding affinity test using phages having sequence was confirmed through ELISA assay. Two peptide sequences (M1 (NCBI accession no. ON493495), M3 (NCBI accession no. ON493496)) expected to specifically bind to the VDBP-Complex were synthesized to evaluate the binding affinity to VDBP-Complex using magnetic beads. Each of the two peptide sequences was conjugated to the surface of magnetic beads, and treated with VDBP-Complex. In addition, to confirm the unique characteristics of this sequence, the binding affinity for VDBP-Complex through several modified peptides was evaluated using magnetic beads. Three modified peptide motif sequences were conjugated to the surface of magnetic beads, and treated with VDBP-Complex.

Here, we report the newly designed phage display technique capable of screening sequences that bind to a target more specifically and selectively. This includes the step of removing the phage that attaches to the control group. Using a new phage display technique, a sequence specifically binding to the VDBP-Complex was screened, and the binding affinity was confirmed by conjugating the peptide sequence (M3) with magnetic beads. It is expected that the M3 peptide sequence discovered using the newly designed phage display technique will show the possibility of determining the serum concentration of VD in complex form by screening the VDBP-Complex, excluding VDBP not bound with VD, in the detection of VD.

## Results

### Coating of VDBP-Complex and VDBP

**Figure 1:**
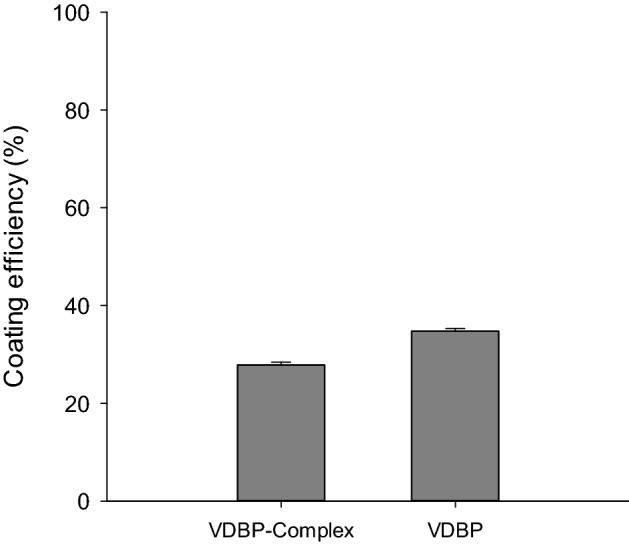
Coating efficiency of VDBP-Complex and VDBP on petri dish. The coating efficiency (%) of VDBP-Complex and VDBP. Each material concentration before and after the coating was measured by Bradford assay, and the coating efficiency was calculated and presented.

VDBP-Complex and VDBP were coated with 0.1 M NaHCO_3_ (pH 8.6) on a petri dish. After the coating step, to confirm the remaining VDBP-Complex and VDBP, the protein concentration of the supernatant was measured using the Bradford assay. Figure 1 shows that it was confirmed that about 27.85% of VDBP-Complex and 34.76% of VDBP were coated. Concentrations of each vitamin-related material were measured by Bradford assay under the same conditions in triplicate. In the VDBP-Complex, the concentration measured before the coating was (2.8340 ± 0.0481) mg/mL, and the concentration of the supernatant remaining after coating was (2.0447 ± 0.0437) mg/mL. In the VDBP, the concentration measured before the coating was (3.1409 ± 0.0565) mg/mL, and the concentration of the supernatant remaining after coating was (2.0491 ± 0.0413) mg/mL (data not shown).

### Confirmation of the added method in phage display technique to screen reliable peptide sequence with high affinity to vitamin-related biocomplex

To select the phage bound against the vitamin-related materials, a Ph.D.-12™ Phage Display Peptide Library was used, which contains a combinatorial library of random 12-mer peptides fused to a pIII protein of M13 phage. As a negative control, a petri dish (polystyrene) with no contents was prepared.

To screen the phages attached to the VDBP-Complex, phage display was performed using 3 different methods. The 1 × 10^9^ of random phages were attached to the VDBP-Complex by biopanning steps for each method. The unbound phages were removed by washing, and the bound phages were eluted, neutralized, and amplified to perform the next round of biopanning. Three rounds of biopanning were performed in the traditional method, four rounds of biopanning were performed in the pre-binding method, and seven rounds of biopanning were performed in the after-binding method. Generally, in the phage display technique, overlapping sequences begin to appear in 3rd biopanning round. In this study, while the pre-binding phage display method and the after-binding phage display method were performed separately, overlapping sequences began to appear in the 3rd biopanning round. Only when using the traditional phage display method did all sequences appear randomly in the 3rd biopanning round. For these reasons, it was judged that it was difficult to screen the target-specific sequence in the traditional phage display method, so only the 3rd biopanning was performed. The overall process including the newly introduced method for biopanning is shown in Supplementary Fig. S2 online. During the pre-binding phage display method, a control-related sequence (target-unrelated sequence) appeared in the 4th biopanning round and the experiment was stopped. During the after-binding phage display method, overlapping sequences began to appear gradually, and it was also confirmed that there was no fast or slow propagating peptide and proceeded until the 7th biopanning round. Table 1 shows the titration data of the biopanning results by each different method. The amount of phage recovered was approximately (10^5^–10^7^) pfu/mL, and the amount of amplified phage was about (10^10^–10^12^) pfu/mL. While the amount of phage to be amplified may have different tendencies depending on the condition of the amplifying step time, the amount of phage to be recovered is the amount of eluted phage compared to the same input (1 × 10^9^ pfu/mL).

**Table 1:** Titration data of the recovery and amplified amount of phage after each phage display method (unit : pfu/mL).

Target	Selection round	Input	Traditional phage display method		Pre-binding phage display method		After-binding phage display method	
			Recovery	Amplified	Recovery	Amplified	Recovery	Amplified
VDBP-Complex	1	1 × 10^9^	4.20 × 10^5^	8.30 × 10^11^	1.70 × 10^7^	5.55 × 10^11^	1.65 × 10^7^	4.95 × 10^11^
	2		8.80 × 10^6^	1.00 × 10^10^	1.76 × 10^6^	2.95 × 10^11^	2.05 × 10^6^	9.05 × 10^11^
	3		1.80 × 10^6^		7.00 × 10^6^	1.30 × 10^12^	2.30 × 10^5^	2.00 × 10^12^
	4				1.50 × 10^5^		1.10 × 10^6^	7.75 × 10^11^
	5						1.12 × 10^6^	4.80 × 10^11^
	6						6.80 × 10^5^	1.75 × 10^12^
	7						2.75 × 10^5^	
VDBP	1	1 × 10^9^	3.40 × 10^5^	7.10 × 10^11^	1.55 × 10^7^	4.20 × 10^11^	3.30 × 10^7^	9.00 × 10^11^
	2		6.40 × 10^6^	4.80 × 10^10^	1.73 × 10^6^	7.35 × 10^11^	2.30 × 10^6^	6.95 × 10^12^
	3		1.22 × 10^6^		5.50 × 10^6^	1.40 × 10^12^	5.95 × 10^5^	7.20 × 10^10^
	4				4.00 × 10^5^		4.80 × 10^5^	9.50 × 10^11^
	5						9.45 × 10^5^	4.10 × 10^11^
	6						5.50 × 10^5^	1.40 × 10^12^
	7						3.95 × 10^5^	
Negative control	1	1 × 10^9^	6.80 × 10^5^	9.75 × 10^11^	9.80 × 10^6^	4.80 × 10^11^	9.30 × 10^6^	6.00 × 10^11^
	2		8.20 × 10^6^	5.75 × 10^10^	1.50 × 10^5^	2.70 × 10^11^	2.50 × 10^6^	1.10 × 10^11^
	3		1.62 × 10^6^		1.80 × 10^6^	1.10 × 10^12^	6.45 × 10^5^	2.00 × 10^11^
	4				1.20 × 10^5^		1.57 × 10^6^	9.00 × 10^11^
	5						1.05 × 10^6^	6.00 × 10^11^
	6						4.50 × 10^5^	1.00 × 10^12^
	7						2.15 × 10^5^	

Summary of data calculated of the amount of phages recovered and amplified by the addition of the pre-binding method and after-binding method, compared to the existing phage display technique. For vitamin D binding protein complex (VDBP-Complex), vitamin D binding protein (VDBP), and empty petri dish (polystyrene used as negative control; NC), 3 rounds of biopanning were performed using the traditional method, 4 rounds using the pre-binding method, and 7 rounds using the after-binding method. The phage titer (pfu/mL) after each round was measured.

*pfu/mL* plaque forming unit per milliliter; *Negative control* target uncoated plate (empty petri dish).

As a result of the titration of output phage recovery in the 2nd round, in the case of the pre-binding method, it could be confirmed that the recovery amount of phage decreased by 20.00% to be about 80.00% in the VDBP-Complex-coated plate, and by 27.03% to be about 72.97% in the VDBP-coated plate, compared with that in the case of the traditional method (Table 2). This showed that the process of preferentially removing the phages attached to the control (petri dish (polystyrene) and BSA) added in the pre-binding method took place efficiently. And, in the 3rd round, it was confirmed that the recovery amount of phage in the after-binding method decreased by 3.29% to about 96.71% in the VDBP-Complex-coated plate, and by 10.82% to about 89.18% in the VDBP-coated plate, compared with that in the case of the pre-binding method (Table 2). This showed that the process of removing the phages that were more selectively attached to the control, among the phages attached to the target added in the after-binding method, has been accomplished.

**Table 2:** Comparison of the recovery amount of each round when using each phage display method (recovery unit: pfu/mL).

	Target	Recovery		Phage recovery reduction rate (%)
		Traditional	Pre-binding	
In the 2nd round	VDBP-Complex	8.80 × 10^6^	1.76 × 10^6^	80.00*
	VDBP	6.40 × 10^6^	1.73 × 10^6^	72.97*
	Negative control	8.20 × 10^6^	1.50 × 10^6^	81.71*

	Target	Recovery		Phage recovery reduction rate (%)**
		Pre-binding	After-binding	
In the 3rd round	VDBP-Complex	7.00 × 10^6^	2.30 × 10^5^	96.71**
	VDBP	5.50 × 10^6^	5.95 × 10^5^	89.18**
	Negative control	1.80 × 10^6^	6.45 × 10^5^	64.17**

*Comparison of the phage recovery amount in the 2nd round when using traditional phage display and pre-binding phage display. The decrease rate of phage recovery amount in the 2nd round when using pre-binding phage display compared to traditional phage display is shown on the right side of the table. When the output pfu/mL of phage (recovery) is set to 100% in the traditional method, it is the value expressed in percent (%) for the recovery reduced in the pre-binding method.

**Comparison of the phage recovery amount in the 3rd round when using the pre-binding phage display and after-binding phage display. The decrease rate of phage recovery amount in the 3rd round when using the after-binding phage display compared to the pre-binding phage display is shown on the right side of the table. When the output pfu/mL of phage (recovery) is set to 100% in the pre-binding method, it is the value expressed in percent (%) for the recovery reduced in the after-binding method.

*VDBP-Complex* Vitamin D binding protein complex, *VDBP* Vitamin D binding protein, *Negative control* empty petri dish (polystyrene).

All phage sequences were analyzed by Bioneer (Korea). The latest two biopanning data were used in each of the three different phage display methods. Phage sequence analysis was performed using output phage titration plates for each method and each biopanning. Individual phage amplification was performed through ER2738 by taking random phage from the blue plaque of each plate, and from them, isolating their DNA; then, PCR was performed with all isolated DNA to amplify. In the traditional method, all the phage sequences appeared randomly, so the most recent biopanning (the 2nd panning and the 3rd panning) sequence data were used. An experimental process was added to find an improvement for the next experimental method. In the pre-binding method, the sequence appearing in the control appeared in the VDBP-Complex-coated plate and VDBP-coated plate. And the most recent biopanning (the 3rd panning and the 4th panning) sequence data were used, and an experimental process was added to find an improvement for the next experimental method. In the after-binding method, sequence data of the 6th panning and 7th panning, the most recent biopanning, were used.

Table 3 presents all the sequences using the traditional method. As a result, none of the overlapping sequences appeared in the 2nd and 3rd biopannings. The diversity of the provided M13 phage library was confirmed in the 1st and 2nd biopannings, so the next biopanning was performed. But even though the third biopanning was performed, it was confirmed that all phage sequences appeared randomly (Table 3). Therefore, it was considered that it was difficult for the traditional phage display method to find overlapping sequences, and the experiment was carried out by adding a new method to progress the pre-binding method.

**Table 3:** DNA sequences analysis data from the two latest biopannings using each phage display method.

Traditional phage display method											
BSA (Negative control)				VDBP-Complex				VDBP			
2nd panning		3rd panning		2nd panning		3rd panning		2nd panning		3rd panning	
Sequence	Freq	Sequence	Freq	Sequence	Freq	Sequence	Freq	Sequence	Freq	Sequence	Freq
ETNTAGHTSLES	1	DTYSHQMKIRVP	1	AIPIWTISEVSL	1	ATFPPINSRTPA	1	APLPSDRSMNPS	1	AHASDRPSQHRV	1
FSPHNLTYNMDA	1	HHGLYRMPVTIE	1	ESWQPVHGLIPL	1	FETTYMYIKSNP	1	ATFNSQFFSKKG	1	AQPLSVYEMDPK	1
GVTDPFFDQHAE	1	HLSYDRSVLLPT	1	FEDSDAFRKFTM	1	FKTPDDSLWPHA	1	DPHWASLLDSVS	1	DDIRPQLSYHGR	1
LSPLSPPMRPLK	1	LPPHAARTPSEF	1	FHSRMLPGRLVP	1	FPLSLGSVSPLN	1	FHEIHTMPLRYA	1	HLTATELANSYH	1
LVAPLDSTAPVL	1	MHPSTSWLDSTP	1	FNSISDAGTGCT	1	GIYPFAQSSTYP	1	HHSLIPPSPVAW	1	HNSGILRTMGAY	1
…	…	…	…	…	…	…	…	…	…	…	…
WVNNSLATPYMS	1	STVGPMSTLNRS	1	SNPFALPISTQD	1	TNPLDARFHEPT	1	TNYIYRYSVDNQ	1	TSGTIFYGNSDV	1
YAPHLSTMLQYH	1	TSSAQLRHGPLL	1	TSLHGDPFHRMH	1	TNQSSQHVLIKE	1	VLAKQHSSVPLQ	1	TTSRVPDNIRLT	1
YDTPNNYFINYY	1	WPDLVHTSDSRT	1	WSTERYSATRYI	1	TSLPFPLASRHA	1	WPNAAPSGADSP	1	TYTLMNPSAMPQ	1
YSSPLMNDAKFP	1	YPVRAVPNQSGQ	1	YLDPVPKANIWL	1	YPDPLIESPKLG	1	WTPDCTLSWISS	1	YPSSVHVQWKLL	1

Pre-binding phage display method											
BSA (Negative control)				VDBP-Complex				VDBP			
3rd Panning		4th Panning		3rd Panning		4th Panning		3rd Panning		4th Panning	
Sequence	Freq	Sequence	Freq	Sequence	Freq	Sequence	Freq	Sequence	Freq	Sequence	Freq
ASSAYLKSMDPA	4	SGVYKVAYDWQH	7	SGVYKVAYDWQH	9	SGVYKVAYDWQH	7	GLHTSATNLYLH	6	GLHTSATNLYLH	2
SGVYKVAYDWQH	4	GLHTSATNLYLH	3	GLHTSATNLYLH	3	GLHTSATNLYLH	4	SGVYKVAYDWQH	4	SLDGAGAALRTS	2
ATDFLPYYHGLL	1	TGAPPRLDARPA	1	AFHPR*METQMY	1	GLHTPIPFVVPFYCH	1	DSQFNKYSIATV	3	DRWVARDPASIF	1
DSQFNKYSIATV	1	ASSAYLKSMDPA	1	GLHTSIPFVVPFYCH	1	SGVYTIPLVVPFYSH	1	GQSEHHMRVASF	2	GDGNSVLKPGNW	1
GDGNSVLKPGNW	1	HTAHVQADRPTQ	1	GSAPLLTVDTSK	1	SLDGAGAALRTS	1	ASSAYLKSMDPA	1	HTPMSSRLSTAS	1
GLHTSATNLYLH	1			…	…	T*TVSTENSKWW	1	…	…	…	…
GSAPLLTVDTSK	1			QWNWPVRSVANV	1			GIATMPPTFSKQ	1	SGVYKVAYDWQH	1
SGALHKSWYAGP	1			SLDGSGAALRTS	1			RTPEMTSLMAWG	1	SKGDSLPFPFAT	1
				SNVPQVPVMGHY	1			VVSPDMNLLLTN	1	SNSIDKVNRPIN	1
				SPFPGVMVHKNN	1			VVSRLPYDRVEA	1	VVSPDMNLLLTN	1

After-binding phage display method											
Petri dish (Negative control)				VDBP-Complex				VDBP			
6th Panning		7th Panning		6th Panning		7th Panning		6th Panning		7th Panning	
Sequence	Freq	Sequence	Freq	Sequence	Freq	Sequence	Freq	Sequence	Freq	Sequence	Freq
YEFHPMGNPLHR	21	YEFHPMGNPLHR	21	SLFTKQYDYFDT	5	SLFTKQYDYFDT	6	TGSAKFLQRDTH	3	TGSAKFLQRDTH	3
SYPSNALSLHKY	1	HDPRMEHSLPKS	5	SFTKTSTFTWRD	2	VPTTSHRVAVLS	3	AFADGYSARRNL	1	ATWWQPDARGTP	1
GTGGVHPATKLT	1	NTTYPTVYADKS	1	AMPPTDLELHSK	2	FSPQNHKPNPVT	2	AFKPTSGLAKLS	1	ATYQNWTLPHRV	1
HDPRMEHSLPKS	1	SYWYEASSYTGV	1	ANGTAHSTPLLW	2	AMPPTDLELHSK	1	AWRPFPSATSGP	1	AWRPSSASTLWN	1
ISAKPIPISMRN	1	TNENLMVRLTHA	1	DRPAHGILEASL	2	IPQRYAPVSNLP	1	FAPYNNLSDNYP	1	GSAARTISPSLL	1
…	…			FSPQNHKPNPVT	1	…	…	…	…	…	…
SYPSNALSLHKY	1			IPQRYAPVSNLP	1	NNSHYYRNIFYT	1	YHGQISANAHGW	1	SPAKPHSFYTGS	1
TASSINLHAAHE	1			…	…	SSAPSMVMSTLF	1	YSSIAPSISNAL	1	SVPLNSWSIFPR	1
TLNVPPAKRSLS	1			NNSHYYRNIFYT	1	SWNHAGQPLTVV	1	YSSTSYRALTLG	1	TADVFSSSRYTR	1
TSLSTAHPMLYQ	1			TSTLYTRAQLWN	1	SYPSNALSLHKY	1	YTSLPTEATDRT	1	VQFTPRSYQPIY	1

DNA sequence analysis was performed from the 40 clones for each biopanning. In the pre-binding phage display method, the underlined sequences indicate a sequence that appears simultaneously in the negative control and VDBP-Complex or VDBP. These sequences were ordered by the highest frequency appearance and significance.

*Freq* Frequency.

Table 3 presents all of the sequences using the pre-binding method. As a result, overlapping sequences are shown, but it was confirmed that most of the overlapping sequences obtained from the VDBP-Complex and VDBP-coated plates were sequences from the control. Also, the diversity of the provided M13 phage library was confirmed in the 1st and 2nd biopannings, so the next biopanning was performed. From the 3rd biopanning, four or more overlapping sequences began to be seen, and it was confirmed that the overlapping sequences appeared in the 4th biopanning (Table 3). Out of the four overlapping sequences that appeared in the control, there were two notable sequences: SGVYKVAYDWQH and GLHTSATNLYLH. The sequence of SGVYKVAYDWQH appeared 9 times in the VDBP-Complex-coated plates, and 4 times in the VDBP-coated plates in the 3rd biopanning (see Supplementary Fig. S1a online). In addition, it appeared 7 times in a VDBP-Complex-coated plate, and once in a VDBP-coated plate in the 4th biopanning, but in the end, it was confirmed that it was equally duplicated on the negative control (Table 3). The sequence of GLHTSATNLYLH appeared 3 times in the VDBP-Complex-coated plate, and 6 times in the VDBP-coated plate in the 4th biopanning (see Supplementary Fig. S1a online). Although it was duplicated 4 times in the VDBP-Complex-coated plate and twice in the VDBP-coated plate in the 4th panning, in the end, it was confirmed that it was equally duplicated on the negative control (see Supplementary Fig. S1b online). Therefore, it was also considered to be difficult for the pre-binding phage display method to find overlapping sequences attaching selectively to the VDBP-Complex and VDBP. So, the experiment was carried out by adding a new method to progress the after-binding method.

Table 3 presents all the sequences using the after-binding method. As a result, overlapping sequences were shown, and it was confirmed that sequences that appear overlapping in the VDBP-Complex and VDBP-coated plate did not appear in the negative control (Table 3). In other words, it can be considered that the after-binding method phage display performed well. At this time, the diversity of the provided M13 phage library was confirmed in the 1st biopanning, so the next biopanning was performed. From the 4th biopanning, noticeable overlapping sequences began to appear. Therefore, the after-binding method can preferentially remove phages that are more selective to control in the 10^13^ pfu/mL phage library compared to the aforementioned methods. An easy-to-understand illustration of how the pre-binding phage display method or the after-binding phage display method works is provided in Supplementary Fig. S3 online.

### VDBP-Complex specific random phage selection by biopanning

To select the phages against VDBP-Complex, the random phage pool from the Ph.D.-12™ Phage Display Peptide Library was attached to VDBP-Complex by the 1st–7th biopannings. From the Ph.D.-12™ Phage Display Peptide Library, phages selectively attached to the petri dish (polystyrene) and BSA surface were first removed (Pre-binding method). The 1.5 mL of 1 × 10^9^ of random phages were first attached to the petri dish (polystyrene). Then, the supernatant was recovered and attached to the BSA-coated plate. Then, the supernatant was recovered and attached to the VDBP-Complex coated plate for each biopanning. The unbound phages were removed by washing with 0.1%.v/v TBST (1–5 round) and 0.3%.v/v TBST (6–7 round). Then, the bound phages were eluted, and recovery of the phages was calculated by titering (Table 1; after-binding phage display method, Recovery). The eluted phages were amplified via ER2738 to perform the next rounds of biopanning, which was also calculated by titering (Table 1; after-binding phage display method, Amplified). Finally, seven rounds of biopanning were performed. Table 1 shows the titration data for all biopanning results. From the 1st round to the 3rd round, it could be seen that the amount of recovery phage is reduced. This seemed to be the result of the removal of target-unrelated phage in the random phage pool due to the introduction of the pre-binding method and the after-binding method. As biopanning progressed from the 3rd round to the 5th round, it was confirmed that the amount of recovery phage increased slightly by about 10 times, or tended to be similar. In the case of VDBP-Complex, the pfu/mL increased from (2.30 × 10^5^) to (1.10 × 10^6^); in the case of VDBP, it increased from (5.95 × 10^5^) to (4.80 × 10^5^); and in the case of the empty petri dish used as the negative control, it increased from (6.45 × 10^5^) to (1.57 × 10^6^). This means that as biopanning progresses, the amount of phages selectively attached to the materials gradually increases. But in the 6th round, for VDBP-Complex and VDBP including control, it could be seen that the amount of recovery phage decreased compared to the 5th round; this is because the concentration of Tween-20 contained in TBST used for washing was increased from (0.1–0.3) %.v/v. This work was done to isolate the weakly bound phages in a relative sense.

### DNA sequences analysis of screened phages

To check the peptide sequence attached to VDBP–Complex, individual DNA of screened phages after the 7th biopanning was analyzed. For sequence analysis, at least 30 individual phages were each amplified from blue plaques in each output phage titration plate, and DNA was isolated and PCR performed. Table 3 presents the identified sequences expected to be specific to each material during all rounds of biopanning. In the case of the empty petri dish used as the negative control, several duplicated sequences appeared for the first time in the 3rd biopanning, but the YEFHPMGNPLHR (CON1) sequence appeared dominantly as it progressed in the 7th biopanning. The sequence of CON1 was thought to bind strongly to polystyrene (petri dish), because it was found only in the empty petri dish (polystyrene) of negative control, not in VDBP-Complex and VDBP. In the case of VDBP-Complex and VDBP-coated plates, sequences with low frequency were sometimes visible, but did not show a noticeable tendency. Instead, several sequences continued to appear in each biopanning, one or two, but they have not yet appeared as many times as the control group, so attention should be paid to them. There are seven and four notable sequences to take note of in VDBP-Complex and VDBP, respectively.

In VDBP-Complex, the dominant sequence was not observed, but several sequences appeared repeatedly in each biopanning round. It was confirmed that SLFTKQYDYFDT (M1) sequence was observed in the 4th, 5th, 6th, and 7th biopanning. VPTTSHRVAVLS (M2) sequence was observed in the 5th and 6th biopanning. SFTKTSTFTWRD (M3) sequence was observed in the 3rd, 4th, 6th, and 7th biopanning. ANGTAHSTPLLW (M4) sequence was observed in the 3rd, 4th, 5th, and 6th biopanning. AMPPTDLELHSK (M5) and FSPQNHKPNPVT (M6) sequences were observed in the 6th and 7th biopanning (data not shown). In VDBP, also, the dominant sequence was not observed, but several sequences appeared repeatedly in each biopanning round. Among them, the TGSAKFLQRDTH (D1) sequence appeared in each biopanning. The GSAARTISPSLL (D2) sequence appeared in the 3rd, 4th, and 6th biopanning. The SSMPINSPATRQ (D3) sequence appeared in the 4th and 5th biopanning (data not shown). In addition, there were several overlapping sequences, but these were excluded because it was said that they could not specifically bind to VDBP-Complex and VDBP as sequences that appeared in the control group in the previous experiments. Sequences that did not appear overlapped were tested as singlet, and then retested as a triplet with sequences that seemed to have a binding affinity. Although it does not have high frequency, an affinity test was conducted by amplifying individual phages having several prominent sequences in addition to the aforementioned 10 phages.

### Binding affinity test with screened phages using ELISA

The affinity of screened phages that were expected to have prominent binding ability was verified by ELISA. A total of 12 screened phages were used for the binding affinity test (described as 'Phage # : Sequence'.; CON1: YEFHPMGNPLHR; D1: TGSAKFLQRDTH; D2: GSAARTISPSLL; D3: SSMPINSPATRQ; D4: ANTELALANRKH; M1: SLFTKQYDYFDT; M2: VPTTSHRVAVLS; M3: SFTKTSTFTWRD; M4: ANGTAHSTPLLW; M5: AMPPTDLELHSK; M6: FSPQNHKPNPVT; M7: SYPSNALSLHKY). Table 4 shows the data of the 5th–7th panning results according to the frequency of the 12 screened phages mentioned above. VDBP and VDBP-Complex were diluted to 20 µg/mL using 0.1 M NaHCO_3_ in the same manner as in the previous phage display, and then 200 µL was dispensed into a 96-well plate, and coated. In the case of Vitamin D, it was diluted to 200 µM using 100% EtOH, and then 200 µL was dispensed into a 96-well, and coated by recrystallization. After that, the individual screened phages that amplified in the same condition were serially diluted and treated according to the desired concentration. After attaching the phage to the vitamin-related material-coated plates, an Anti-M13 antibody [B62-FE2] (HRP) was added, and 1 h later, ABTS solution was added with 30% H_2_O_2_. The HRP-conjugated Anti-M13 monoclonal antibody facilitates ABTS oxidation in the presence of H_2_O_2_, turning ABTS into a blue-green oxidized product. Therefore, the more phages that were bound to the target, the higher the absorbance value that could be checked. Therefore, ELISA is a useful method to determine the relative affinity of the screened phages.

**Table 4:** DNA sequences of the 120 clones for each material-specific binding phage using the after-binding method.

The 5th–7th biopannings in the after-binding method								
Negative control			VDBP			VDBP-Complex		
Phage #	Sequence	Frequency	Phage #	Sequence	Frequency	Phage #	Sequence	Frequency
CON1	YEFHPMGNPLHR	49	D1	TGSAKFLQRDTH	10	M1	SLFTKQYDYFDT	15
CON2	HDPRMEHSLPKS	10	D2	GSAARTISPSLL	4	M2	VPTTSHRVAVLS	6
CON3	NTTYPTVYADKS	4	D3	SSMPINSPATRQ	3	M3	SFTKTSTFTWRD	4
		1	D4	ANTELALANRKH	1	M4	ANGTAHSTPLLW	4
		:			:	M5	AMPPTDLELHSK	3
		:			:	M6	FSPQNHKPNPVT	3
		1			1	M7	SYPSNALSLHKY	1
Total		120	Total		120	Total		120

From the 5th to the 7th biopanning, the DNA sequence of a total of 120 clones for each material-specific biding phage using the after-binding method was analyzed. Forty phage clones were picked for each biopanning. These sequences were ordered by the highest frequency appearance and significance. Phage # has been renumbered by composing this table.

**Figure 2:**
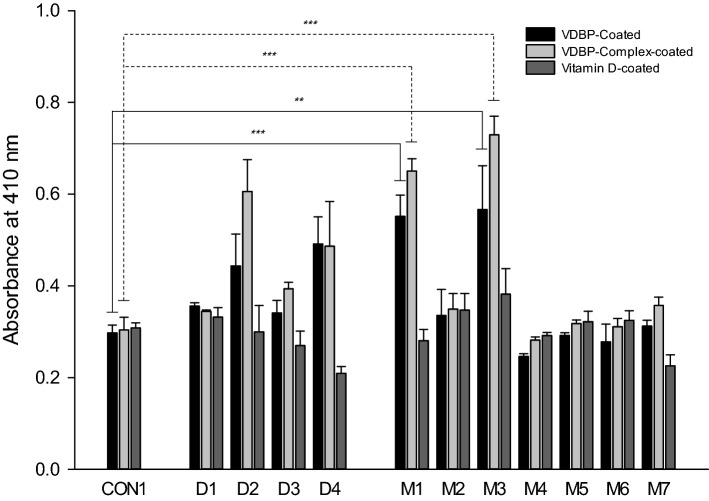
Binding affinity ability of each phage clone screened. ELISA assay for each of the screened phage clones having 12 sequences to VDBP, VDBP-Complex, VD for binding affinity test. The M3 (SFTKTSTFTWRD) sequence shows the highest affinity, while the M1 (SLFTKQYDYFDT) sequence shows the second highest affinity in this result. Data are expressed as the means of the absorbance value at (410 ± SD) nm (n = 3). **p < 0.01 and ***p < 0.001 vs. CON1.

Figure 2 shows the ELISA results. In the case of the CON1 phage with the YEFHPMGNPLHR sequence used as a negative control, it was confirmed that each of the targets (VDBP, VDBP-Complex, VD) had absorbance values of (0.298 ± 0.017), (0.304 ± 0.0276), and (0.308 ± 0.011), respectively. It was confirmed that the majority of the 12 selected phages did not have a value of over 0.6. Phages with absorbance values exceeding 0.6 were D2 (GSAARTISPSLL) sequence, M1 (SLFTKQYDYFDT) sequence, and M3 (SFTKTSTFTWRD) sequence. In the case of D2 phage, it has a value of (0.444 ± 0.069) in the VDBP-coated plate, (0.606 ± 0.069) in the VDBP-Complex-coated plate, and (0.300 ± 0.057) in VD-coated plate. But, D2 was a phage having a sequence that only appeared overlapping in the VDBP-coated plate, and the phage could be seen to have a low absorbance value in the VDBP-coated. In the case of M1 phage, the values were (0.552 ± 0.046) in the VDBP-coated plate, (0.650 ± 0.027) in the VDBP-Complex-coated plate, and (0.281 ± 0.024) in the VD-coated plate. So, the value of (0.650 ± 0.027) in VDBP-Complex seemed to have significant meaning. In the case of M3 phage, the values were (0.566 ± 0.096) in the VDBP-coated plate, (0.729 ± 0.041) in the VDBP-Complex-coated plate, and (0.382 ± 0.056) in the VD-coated plate. So, the value of (0.729 ± 0.041) in VDBP-Complex seemed to have significant meaning. For VDBP-Complex, the sequences of the M3 phage had the highest binding affinity, and the sequences of the M1 phage had the second-highest binding affinity. Therefore, the subsequent experiment proceeded to two peptides (M1, M3) with these sequences.

### VDBP-Complex concentration decrease test using synthesized peptides

**Figure 3:**
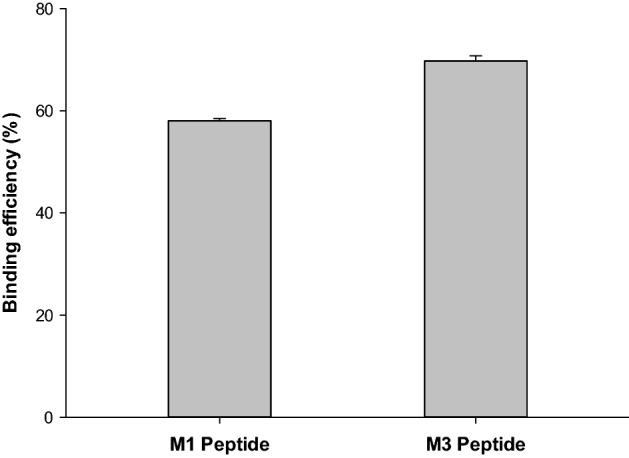
Binding efficiency of two synthesized peptide sequence selected from phage with high affinity to VDBP-Complex. Binding efficiency of two peptide sequences (M1, M3) selected from phage with high affinity to VDBP-Complex. The binding affinity of the phage clones to VDBP-Complex was confirmed through ELISA assay, and a peptide sequence having a high affinity among them was synthesized, and conjugated with magnetic beads. The VDBP-Complex concentration decrease was measured by Bradford assay of the remaining supernatant, after removing the magnetic beads by magnetic field.

To study the role of two VDBP-Complex specific peptides selected from sequences analysis and binding affinity ELISA using phage with the corresponding sequence, the M1 and M3 peptides were conjugated to magnetic beads. Table 5 shows these sequences. Since the ELISA result shows the phage with the M3 sequence has the highest absorbance value, the M3 sequence has the highest potential for binding to VDBP-Complex. The conjugation of the M3 peptide and magnetic beads was performed according to some steps of the Dynabeads™ M-270 Amine protocol. Peptide-conjugated beads were treated with VDBP-Complex, and then removed by a magnetic field. After that, their concentration was measured by Bradford assay. Figure 3 shows the results. It was confirmed that the concentration was decreased to [(2.6187 ± 0.002) and (1.8901 ± 0.006)] µg/mL when M1 and M3 peptide-conjugated beads were treated for VDBP-Complex at a concentration of (6.2410 ± 0.003) µg/mL, respectively. The binding efficiency of the M1 peptide-conjugated beads was about 58.06%, while the binding efficiency of the M3 peptide-conjugated beads was about 69.72%. The fluorescence of FAM located in the N-terminal of the peptide was used to confirm the conjugation rate of the beads with peptide. Of the 50 µg/mL peptide, 29.78 µg/mL of the peptide was bound, and it was confirmed that about 59.39% of the peptide was attached to the beads (data not shown). As a result, the M3 sequence had the highest affinity to VDBP-Complex.

**Table 5:** Peptide sequences modification: SFT (Ser-Phe-Thr) motifs using C1 as the backbone for checking characteristic.

Peptide name	Sequence	Modification description
C1*	FAM—*Y E F H P M G N P L H R*	(Backbone of the sequence to be modified)
M1*	FAM—**S** L **F T** K Q Y D Y **F** D **T**	(Peptide sequence that is expected to specifically bind to VDBP-Complex)
M3*	FAM—**S F T** K T S T **F T** W R D	(Peptide sequence that is expected to specifically bind to VDBP-Complex)
Mod1	FAM—**S** *E F H P M G* **F T** *K H R*	Based on the C1 sequence, with the Ser (S) in the first position, and the Phe-Thr (F-T) sequence at the back
Mod2	FAM—**S F T** *K P M G N P L H R*	Based on the C1 sequence, with the Ser (S) and Phe-Thr (F-T) parts in front
Mod3	FAM—**S F T** *H* **S F T** *N* **S F T** *R*	Based on the C1 sequence, with the Ser (S), Phe (F), and Thr (T) sequences appearing repeatedly

FAM labeling modification is to calculate the amount of the peptide immobilized on the magnetic beads using fluorometer. * indicates control-specific (C1) and target-specific (M1, M3) sequences obtained through biopanning. No modification other than FAM labeling at the n-terminus was performed. Mod1, Mod2, and Mod3 sequences were modified based on the C1 sequence.

Significant values are in bold.

### VDBP-Complex specific peptide characteristic

**Figure 4:**
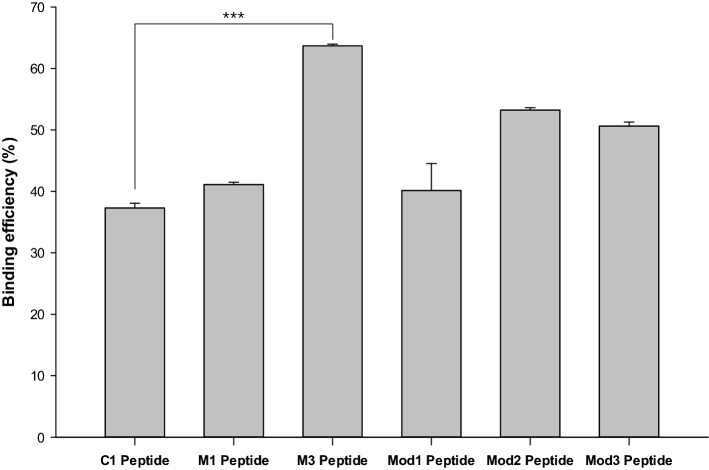
Unique binding affinity ability of the M3 peptide sequence to VDBP-complex. Binding efficiency of one control peptide sequence (C1), two peptide sequences (M1, M3) having high affinity to the VDBP-Complex, and three modified peptide sequences (Mod1, Mod2, and Mod3). The peptide sequences having a high affinity to the control or the VDBP-Complex, respectively, were synthesized, and conjugated with magnetic beads (C1, M1, and M3). Using the C1 sequence as a backbone, one sequence in which the S (Ser) is in front and the F-T (Phe-Thr) part wass in the back (Mod1); one sequence in which the S (Ser) and F-T (Phe-Thr) part appeared in front (Mod2); and one sequence in which the S (Ser), F (Phe), and T (Thr) sequences appeared repeatedly (Mod3), were customized. These three peptide sequences were synthesized and conjugated with magnetic beads. The VDBP-Complex concentration decrease was measured by Bradford assay of the remaining supernatant, after removing the magnetic beads by magnetic field. A p value < 0.001 was considered significant [(protein concentration ± SD) in µg/mL, n = 6]. ***p < 0.001 vs. C1 Peptide.

A modified peptide was used to confirm that the sequence was specifically binding to the VDBP-Complex. The modified peptide was partially modified using the control-specific peptide sequence (C1; YEFHPMGNPLHR) as a backbone. The peptide immobilized on the magnetic beads was confirmed by fluorometer by modifying the N-terminal of the peptide with the FAM label, which is a fluorescent dye. Table 5 presents all peptide sequences. S (Ser), F (Phe), and T (Thr) were noticeably repeated in the M1 and M3 peptide sequences, and three modified peptides were customized to check whether the sequence order was affected. Using the C1 peptide sequence as a backbone, one sequence, in which the Ser (S) was in front and the Phe-Thr (F-T) sequence was in the back (Mod1); one sequence, in which the Ser (S) and Phe-Thr (F-T) parts appeared in front (Mod2); and one sequence, in which the Ser (S), Phe (F), and Thr (T) sequences appeared repeatedly (Mod3), were customized. The conjugation of all the peptides with magnetic beads was performed according to some steps of the Dynabeads™ M-270 Amine protocol. Peptide-conjugated beads were treated with VDBP-Complex, and then removed by a magnetic field. After that, their concentration was obtained by Bradford assay. When VDBP-Complex was treated at a concentration of (2.7505 ± 0.022) µg/mL, it was confirmed that the concentrations of VDBP-Complex were decreased to [(1.696 ± 0.016), (1.594 ± 0.008), (0.983 ± 0.006), (1.619 ± 0.094), (1.265 ± 0.008), and (1.335 ± 0.013)] µg/mL by C1, M1, M3, Mod1, Mod2, and Mod3 peptide-conjugated beads, respectively. It was confirmed that all peptides were coated on the magnetic beads by about (54.71 ± 1.34) % (data not shown). Figure 4 shows the result of confirming the VDBP-Complex reduction rate based on this. In the case of C1 peptide-conjugated beads used as a control, the VDBP-Complex reduction rate was (37.30 ± 0.76) %. In the case of the M1 peptide-conjugated beads and the M3 peptide-conjugated beads, VDBP-Complex reduction rates of [(41.10 ± 0.40) and (63.67 ± 0.26)] % were confirmed, respectively. In the case of the modified peptide sequence-conjugated beads (Mod1, Mod2, and Mod3), it was confirmed that the VDBP-Complex reduction rates were [(40.14 ± 4.38), (53.23 ± 0.39), and (50.63 ± 0.63)] %, respectively. It was confirmed that the M3 peptide-conjugated beads had the highest rate of VDBP-Complex reduction, which is consistent with Result 2.6. The M1 peptide-conjugated beads and Mod1 peptide-conjugated beads showed VDBP-Complex reduction rates that were similar to those of the C1 peptide-conjugated beads; meanwhile, the Mod2 peptide-conjugated beads and Mod3 peptide-conjugated beads showed VDBP-Complex reduction rates that were less than 1.4 times that of the control group.

## Discussion

Recently, Vitamin D has been in the spotlight in many areas as well as enhancing immunity. In detecting vitamin D, interest in detecting the vitamin D bind protein complex (VDBP-Complex) is increasing rather than directly detecting vitamin D (VD)^1,11^. This is because VD is in a complex form by binding vitamin D binding protein (VDBP) in the blood^10^.

In this study, a newly designed phage display technique was used to screen and identify the VDBP-Complex specific peptide for detecting the VDBP-Complex containing VDBP, the protein with a size of 55 kDa. In the traditional phage display method, the phages that bind to the target appear randomly, even when biopanning is performed several times. The traditional phage display method has the disadvantage of poor selection efficiency by simply relying on batch equilibrium adsorption/desorption. It may reduce the binding efficiency of target specific phages as well as increase unnecessarily repeated screening rounds^23^. Therefore this makes it difficult to screen phages that selectively bind only to the target. To overcome this, two new processes were added to the traditional phage display technique, allowing more selective sequences to be identified in the target. The first experimental method added is to bind the phage firstly to a petri dish (polystyrene) and BSA used as control groups, and then bind the unbound phage pool to the target (vitamin related biocomplex). After adding this method, it was confirmed that overlapping sequences appeared through DNA analysis, but the overlapping sequences from the target-coated plate were those that appeared in the control group. Thus, the second experimental method added was to bind the eluted phage pool, which was bound to the target, to the petri dish again, and to collect only the unbound phage (supernatant). After adding this method, it was confirmed that overlapping sequences appeared through DNA analysis, and that sequences appearing in the control group did not appear in the target-coated plate.

In brief, when the traditional phage display method is used, it could be confirmed that all sequences appear randomly. The reason is expected to be because it has a diversity of more than 4000 trillion (≤ 20^12^), due to the random 12-mer peptide of the M13 phage display 12-mer-peptide library (Ph.D.-12). It makes it difficult to find only phage selective for the target. When using the pre-binding phage display method with the addition of the experimental method, the overlapping sequence appeared, but it also appeared in the control group simultaneously. When using the after-binding phage display method with the addition of the experimental method, overlapping sequences appeared, and the sequences in the control group did not appear in VDBP-Complex and VDBP. Hence, the phage display technique with the addition of these experimental procedures will be a new way to find phages selective to the target.

With this revised phage display technique, phages that selectively bind to VDBP-Complex were screened through the 7th biopanning. A total of 12 phage sequences that selectively bind to each material were screened and analyzed through DNA analysis. A binding affinity test using a phage with a VDBP-Complex specific sequence by ELISA and VDBP-Complex concentration reduction test using magnetic beads conjugated with the corresponding sequence was performed. One phage sequence (CON1) predominantly shown in the control group, four phage sequences (D1, D2, D3, and D4) expected to specifically bind to VDBP, and seven phage sequences (M1, M2, M3, M4, M5, M6, and M7) expected to specifically bind to VDBP-Complex were prominent phage sequences during the 7th biopanning. The results of ELISA showed that 2 phages (M1; SLFTKQYDYFDT and M3; SFTKTSTFTWRD) of the 12 phages that were expected to bind selectively had a higher binding affinity ability to the VDBP-Complex. In the case of the M1 sequence, the binding affinity ability to VDBP-Complex was more than twice as compared to the control. And the binding affinity ability of the M3 sequence was approximately 2.4 times higher than compared to the control. Two peptide sequences (M1, M3) expected to specifically bind to the VDBP-Complex were synthesized to evaluate the binding affinity to VDBP-Complex using magnetic beads. The two-peptide sequence was conjugated to the surface of magnetic beads, and treated with VDBP-Complex^24^. As a result, it was confirmed that the concentration of VDBP-Complex was reduced by 69.72 % using M3 sequence conjugated magnetic beads. A modified peptide (SFT (Ser-Phe-Thr) motif) sequence was used to confirm the unique characteristics of the M3 sequence. It was attempted to determine whether the binding ability is due to the Phe-Thr (F-T) sequence appearing in the front, or whether the binding ability is increased if the Phe-Thr (F-T) sequence is simply duplicated. In conclusion, it was confirmed that all the modified peptides were lower than the binding affinity of the M3 native sequence. These results suggest that the epitope for the VDBP-Complex antibody is not only determined by the repetitive appearance of Ser, Phe, and Thr (S, F, and T). The results also suggest that most of the binding energy for the VDBP-Complex–antibody interaction is contributed by the first 4 residues of the M3 sequence (Ser-Phe-Thr-Lys; S-F-T-K), and that some flexibility is allowed in the 5th position.

Therefore, the M3 sequence is able to specifically bind to the VDBP-Complex, which is expected to be useful to determine the vitamin D concentration in the blood.

## Conclusion

In this paper, M3, a peptide sequence that selectively binds to the VDBP-Complex, was screened and identified. This is the result obtained by the newly designed phage display method by adding two experimental methods (pre-binding and after-binding). This newly designed phage display method is expected to be able to effectively screen phage sequences that are more selectively attached to the target. Two phages that specifically bind to VDBP-Complex were screened through a newly designed phage display technique, and verified by ELISA. An additional binding affinity test was performed by synthesizing the peptide sequences of these two phages. The synthesized peptide sequence was conjugated with magnetic beads, and the peptide-conjugated magnetic beads reduced the concentration of VDBP-Complex by 69.72%. To confirm that it is a unique characteristic of this sequence, a binding affinity test using the modified peptides (SFT (Ser-Phe-Thr) motif) was performed. The modified peptide sequence was conjugated with magnetic beads, and a VDBP-Complex recovery experiment was performed. The modified sequences did not surpass the VDBP-Complex binding ability of the M3 sequence, and this is expected to be the intrinsic VDBP-Complex binding ability of the sequence (M3). Therefore, the SFTKTSTFTWRD (M3) peptide sequence screened using the phage display technique shows the possibility of determining the serum concentration of VD in the complex form by screening the VDBP-Complex, except for VDBP that is not bound with VD, in the detection of VD.

## Material and methods

### Vitamin-related reagents

The vitamin D binding protein complex (VDBP-Complex) was provided by Chungbuk National University (Korea). Its concentration was measured by Bradford assay (Bio-Rad, USA). It should be stored at 4 °C in the refrigerator.

The vitamin D binding protein (VDBP) was provided by MyBioSource (Cat#MBS568853, USA). It was reconstituted with 250 µL of filtered sterile third distilled water as described in the manual. It should be stored at − 20 °C or below. Its concentration was measured by Bradford assay (Bio-Rad, USA).

VDBP-Complex and VDBP were diluted using NaHCO_3_ buffer to proceed with the coating step. Each target was diluted with 0.1 M NaHCO3 (pH 8.6) to a concentration suitable for coating of 20 µg/mL.

Vitamin D (VD) was provided by Merck (Cat#63283-36-3, Germany). It was reconstituted with 238.86 µL 100% EtOH to 10 mM, as described in the manual. It should be stored at − 20 °C.

### M13 phage library and bacterial strains

The M13 phage display 12mer-peptide library (Ph.D.-12), -96 gIII sequencing primer (5′-HOCCC TCA TAG TTA GCG TAA CG-3′), and *Escherichia coli* ER2738[F´*proA*^+^*B*^+^ *lacI*^*q*^* Δ(lacZ)M15 zzf::Tn*10(Tet^R^)/*fhuA2 glnV Δ(lac-proAB) thi-1 Δ(hsdS-mcrB)5*] host strain were provided by New England Biolabs (NEB; Cat#E8110S, USA). All stocks were stored at − 20 °C. All methodology of experiments using the Ph.D.-12 library, including media, solution, and buffer, ER2738 strain maintenance and storage, phage titering, amplification and storage, and ssDNA purification of M13 virus is described in the Ph.D.™-12 Phage Display Peptide Library Kit manual^25^.

### Other materials

Anti-M13 antibody [B62-FE2] (HRP) was used for ELISA, which was provided by Abcam (Cat#ab50370, UK). X-gal (5-bromo-4-chloro-3-indolyl-β-d-galactopyranoside) and IPTG (Isopropyl β-D-1-thiogalactopyranoside) were provided by Cellconic (Cat#F240703, Cat#F091604, Korea). PEG (Poly(ethylene glycol); BioUltra, 8,000) and ABTS (2,2′-Azino-bis(3-ethylbenzothiazoline-6-sulfonic acid) diammonium salt) were provided by Sigma-Aldrich (Cat#89510, Cat#A1888, USA). Quick Start Bradford 1X Dye reagent was provided by Biorad (Cat#500-0205, USA). A petri dish was provided by SPL (Cat#10060, Korea). Nunc™ MicroWell™ 96-Well plate was provided by ThermoFisher (Cat#167008, USA).

### Coating the vitamin-related materials for biopanning

VDBP-Complex and VDBP were prepared by dilution with the concentration of 20 µg/mL with 0.1 M NaHCO3 (pH 8.6; dilution solvent). Petri dishes were coated with 4 mL of VDBP-Complex or VDBP, in solvent dilution respectively, overnight at 4 °C. An empty petri dish with 4 mL of dilution solvent was used as a negative control. Each dish was rocked vigorously overnight at 4 °C. To check if the coating worked, Bradford reagent was used. After coating, each solution was poured off, and 5 mL of third distilled water was added to wash out. Suction using a pipette was applied, and the dish was slapped face down onto a clean paper towel to remove the supernatant clearly. This was repeated 5 times. Then, 1 mL of Bradford 1X dye reagent was added to each dish. Each dish was mixed well using a pipette, and left in a dark condition for 5 min to examine the reaction. Additionally, to confirm the coating efficiency, a Bradford assay was performed. The 50 µL each of the VDBP-Complex solution, VDBP solution, and dilution solvent were put separately in a 96-well plate for the triplicate wells, and the plate rocked vigorously overnight at 4 °C. And Bradford assay was performed with the collected supernatant. Fluorescence was measured by a GloMax^®^ Explorer system (USA).

#### Coating the BSA for pre-binding method

BSA was diluted to a concentration of 5 mg/mL with 0.1 M NaHCO3 (pH 8.6) and coated on the petri dish. Petri dishes were coated with 4 mL of BSA overnight at 4 °C for use in pre-binding. Bradford reagent was used to determine whether the coating was effective. After coating, the solution was poured off, and 5 mL of third distilled water was added to wash out. To remove the supernatant clearly, a pipette was used to apply suction, and the dish was slapped face down onto a clean paper towel. This was repeated 5 times. Then, 1 mL of Bradford 1X dye reagent was added to a petri dish. The petri dish was mixed well using a pipette, and left in a dark condition for 5 min to examine the reaction.

### Phage selection with an affinity for VDBP-Complex including phage titration and amplification

To select the phage bound against the vitamin-related materials, a Ph.D.-12™ Phage Display Peptide Library was used, which contains a combinatorial library of random 12-mer peptides fused to a pIII protein of M13 phage. An empty petri dish (polystyrene) was prepared as a negative control. Since then, all the processes referred to in as described in Material and methods 5.5 are called biopanning.

#### Pre-binding method against control surface (petri dish (polystyrene) and BSA)

One point five mL of phage pool diluted with TBST (TBS + 0.1%.v/v Tween-20) to 1 × 10^9^ Plaque Forming Units per mL (pfu/mL) from the phage library was attached to the petri dish (polystyrene). Then, the solution was rocked at room temperature (RT) for 45 min to remove phages attached to the petri dish (polystyrene) first. The BSA-coated plate as described in Material and methods 5.4.1 was washed 5 times with TBST. Then, the supernatant of the phage pool reacted with a petri dish (polystyrene) and was recovered and put into the BSA-coated plate. Also, it was bound by rocking at room temperature (RT) for 45 min. After that, unbound phages were collected and used for main biopanning. Like this, a method of removing the phages that selectively attach to the petri dish (polystyrene) and BSA used as a control was added and named ‘pre-binding’.

#### Main biopanning I (positive screening against VDBP-Complex)

After the pre-binding method, the solution contained in the VDBP-Complex-coated plate was taken out, and the coated target was washed 5 times with TBST. The recovery phage pool from the pre-binding method was added, and the solution was rocked at room temperature (RT) for 45 min for the binding step. The coated target was washed 5 times with 1 mL of 0.1%.v/v TBST (1–5 round) and 0.3%.v/v TBST (6–7 round) to remove the unbound phages. The bound phage was eluted with 1 mL of an elution buffer [1 mg/mL BSA in 0.2 M Glycine–HCl (pH 2.2)]. The elution mixture was rocked softly for 15 min at RT, then pipetted to collect the eluted phage into a microtube, and neutralized with 150 μL of 1 M Tris–HCl (pH 9.1).

#### After-binding method for removing weak binding phages

All 1.15 mL of neutralized phages were treated in a petri dish (polystyrene), and the solution was rocked at room temperature (RT) for 30 min. Only the supernatant from which the phage strongly bound to polystyrene was removed was collected. The 10 μL of recovered phage was used to titer the phage amount (termed the output phage). Like this, among the phages selective to the target, a method of removing the phages that selectively attach to control was added and named ‘after-binding’. It will be amplified by the inoculation of ER2738 in the same method as a traditional phage display method. The remaining solution was stored at 4 °C.

#### Main biopanning II (amplification of positive screening phage for VDBP-Complex)

To prepare the next biopanning, ER2738 was inoculated in advance with (5–10) mL LB medium (10 g Bacto-Tryptone, 5 g yeast extract, 5 g NaCl) in a conical tube, and incubated at 180 rpm at 37 °C for 20 h. Then, the recovered phages were amplified by adding 500 μL of output phage to the 20 mL LB with ER2738 in a 250 mL Erlenmeyer flask. The culture was incubated at 180 rpm at 37 °C for 4.5 h. Each 1.3 mL of the culture medium was transferred to a total of 6 microtubes, and 1.2 mL of the supernatant was recovered by centrifugation at 12,000*g* at 4 °C for 10 min. Thereafter, performed in the same way for 6 microtubes, unless otherwise noted. Centrifuged again under the same conditions to recover 1 mL of the supernatant. Then, the 1 mL of supernatant was mixed with 200 μL of 20% PEG/2.5 M NaCl, and precipitated at 4 °C for overnight. The day after, the PEG precipitation was centrifuged at 12,000*g* for 15 min at 4 °C. The supernatant was discarded clearly, and the pellet sized fingerprint smear was suspended in 1 mL of TBS, then centrifuged at 14,000 rpm for 5 min at 4 °C to pellet the residual cells. The 800 μL of supernatant was mixed with 200 μL of 20% PEG/2.5 M NaCl, and precipitated at 4 °C for 45 min. Then, centrifuged at 14,000 rpm for 10 min at 4 °C, the supernatant was discarded, and the pellet was suspended in 200 μL of TBS. The 200 μL of TBS from each of the 6 microtubes was pipetted, and gathered to a fresh tube, and centrifuged briefly for about 1 min. The supernatant, which is amplified eluate, was transferred. The amplified phages can be stored at 4 °C. The amplified phage was diluted equal to the input phage (1 × 10^9^ pfu/mL), and the above step was repeated in the next round of biopanning.

The output phages and amplified phages were made by serial 1/10 dilutions for titering. Ten μL of each phage dilution was added to 200 µL of the ER2738 culture grown to an OD_600_ of 0.7, and incubated at RT for 5 min. The infected cells with phage were transferred to a conical tube containing 5 mL of around 40 °C of Top Agar (10 g Bacto-Tryptone, 5 g yeast extract, 5 g NaCl, 7 g Bacto-Agar), and directly poured onto LB/IPTG/X-gal plate (which contained 1 mL of IPTG/X-gal stock (1.25 g IPTG (isopropyl-β-D-thiogalactoside) and 1 g X-gal (5-bromo-4-chloro-3-indolyl-β-D-galactoside) in 25 mL DMF (dimethylformamide)) per 1 L). After standing for 1 h to solidify, the plates were incubated at 37 °C for overnight. The number of blue plaques on each plate was counted, and the pfu/mL value was calculated, considering the dilution factor of the phage.

### Individual phage preparation for phage DNA separation and sequences analysis of screened phage

At least 20 individual phages must be used for the phage DNA sequence analysis, and 30 or more individual phages are recommended to be picked in this study. To amplify individual phage that is bound specifically to VDBP-Complex, ER2738 in the early-log phase (OD600 of (0.01–0.05)) is required, which had been inoculated the night before. It was diluted 1:100 with LB medium. Each 1 mL of the diluted ER2738 culture was dispensed into a microtube. Each blue plaque (individual phage) was taken from the output phage titration plates using the smallest tip (e.g., 10 µL tip), and suspended in each tube. The tubes were incubated at 37 °C for (4.5–5) h with vigorous shaking (180 rpm) for amplification. The culture was centrifuged at 14,000 rpm for 1 min at 4 °C. The supernatant was then transferred to a fresh tube. Only 500 µL of the supernatant was used for phage DNA isolation, so 300 µL of the remaining supernatant was stored at − 70 °C in 50% glycerol stock form for the binding affinity test.

For the individual phage DNA isolation, 200 µL of 20% PEG/2.5 M NaCl was added to 500 µL of the supernatant (individual phage), and the mixture was incubated at RT for 15 min. It was then centrifuged at 14,000 rpm for 10 min at 4 °C, the supernatant discarded, and re-centrifuged briefly, to completely remove the residual supernatant using a pipette. Note that a phage pellet may not be visible, or a very small smear may be seen. Iodide buffer (100 µL) was added to the pellet, and tapped to mix briefly. Then, 250 µL of 100% ethanol was added, mixed briefly, and incubated at RT for 15 min. The mixture was centrifuged at 14,000 rpm for 10 min at 4 °C, the supernatant was poured off, and the pellet was washed with 500 µL of 70% ethanol (stored at − 20 °C). Then, the mixture was re-centrifuged at 14,000 rpm for 10 min at 4 °C, and at this time, the supernatant was removed clearly by pipette. The residue was then dried at RT for (1–2) h to evaporate the remaining ethanol covering it with aluminum foil, until there was no smell of alcohol. After that, the pellet was suspended in 30 µL of TE buffer, and stored at – 20 °C.

After DNA isolation, PCR was performed to amplify the gene of the pIII terminal peptide of phage DNA, and analyze the DNA sequences. The total volume of PCR product was 50 µL, which contains 37.8 µL of TDW, 5 µL of 10X Ex Taq buffer, 4 µL of dNTP, 1 µL of front-primer, 1 µL of reverse-primer, 1 µL of phage DNA template, and 0.2 µL of Ex Taq. 10X Ex Taq buffer, dNTP, and Ex Taq were provided by Takara (Japan). Front-primer (5’-CCG ATT CCT TTA GTG GTA CCT TTC TAT -3’) and reverse-primer (5’-CCC TCA TAG TTA GCG TAA CG-3’) were synthesized by Bioneer (Korea). The 35 cycles of PCR were repeated (Pre-denatured at 95.0 °C for 5 min, Denatured at 95.0 °C for 30 s, Annealing at 53.5 °C for 30 s, and Extension at 72.0 °C for 1 min 30 s), and this was followed by an extension at 72.0 °C for 7 min. After the PCR step, all of the PCR product was purified using the Expin™ PCR SV purification kit, which was provided by GeneAll Biotechnology (Korea). The DNA purification was performed according to some steps of Expin™ PCR SV in the Expin™ Protocol Handbook manual. After completely removing the NW buffer in step 4 of the manual, only the upper column was transferred to a fresh tube, and dried at 37 °C for 30 min. After that, 40 µL of TDW was added, incubated for 1 min, centrifuged for 1 min 30 s, and stored at − 20 °C. All the DNA sequences were analyzed by Bioneer (Korea).

### Affinity test with screened phage (ELISA)

An affinity test was performed with a phage having a sequence expected to specifically bind to VDBP-Complex, which was amplified to at least 10^13^ pfu/mL. In advance, ER2738 was inoculated with (5–10) mL LB in a conical tube, and incubated at 180 rpm at 37 °C for 20 h. ER2738 was inoculated diluted with 1:100 into 20 mL LB medium in a 250 mL Erlenmeyer flask, adding 5 μL individual phage, and incubated at 180 rpm, 37 °C for 4.5 h. After 4.5 h, each 1.3 mL of the culture medium was transferred to a total of 12 microtubes, and 1.2 mL of the supernatant was recovered by centrifugation at 12,000*g* at 4 °C for 10 min. Thereafter, performed in the same way for 12 microtubes, unless otherwise noted. Centrifuged again under the same conditions to recover 1 mL of the supernatant. Then, the 1 mL of supernatant was mixed with 200 μL of 20% PEG/2.5 M NaCl, and precipitated at 4 °C for overnight. The day after, the solution was centrifuged for PEG precipitation at 12,000*g* for 15 min at 4 °C. The supernatant was discarded clearly, and the pellet was suspended in 1 mL of TBS, then centrifuged at 14,000 rpm for 5 min at 4 °C to pellet residual cells. The 1 mL of supernatant was mixed with 200 μL of 20% PEG/2.5 M NaCl, and precipitated at 4 °C for 45 min. It was then centrifuged at 14,000 rpm for 10 min at 4 °C, the supernatant discarded clearly, and the pellet suspended in 50 μL of TBS. Suspended 50 μL of each TBS from each of the 12 microtubes was pipetted, gathered into a fresh tube, and briefly centrifuged for about 1 min. The supernatant was transferred, which is individual amplified eluate. Each individual amplified phage was titrated, and stored at 4 °C. VDBP-Complex and VDBP were diluted with 0.1 M NaHCO3 (pH 8.6) to a concentration of 20 μg/mL, and 200 μL each was added to a 96-well plate (Thermo Fisher Scientific, Cat#167008, USA). Plates were incubated with the target overnight at 4 °C in a humidified container using an agitation machine. A 96-well plate was filled with 200 μL of each 200 μM concentration of vitamin D that had been diluted with 100% EtOH. Plates were incubated overnight at RT to evaporate ethanol before being coated with vitamin D. Recrystallization resulted in the coating of vitamin D. Whether the coating was done or not, it could be seen by the naked eye. The next day, the phage was serially diluted with (10^7^–10^13^) pfu/mL in a new 96-well plate. The coated plate was washed 3 times with TBST (0.1%.v/v), and the phage was transferred from a new plate in which the phage was diluted to the vitamin-related material coated plate. After that, it was reacted in an agitation machine at RT for 1 h and 30 min. Anti-M13 antibody [B62-FE2] (HRP) was used for ELISA, which was provided by Abcam (Cat#ab50370, UK). For the HRP-conjugating, an anti-M13 antibody was diluted 20-fold in PBS containing 0.1% BSA according to the manual. After washing the phage binding plate 3 times with TBST (0.1%.v/v), 100 μL of antibody was added, and reacted with an agitation machine for 1 h. HRP substrate solution (36 μL of 30% H_2_O_2_ was added to 21 mL of ABTS solution) was prepared to check color development about 10 min before washing, after washing 3 times with TBST (0.1%.v/v), 200 μL of HRP substrate solution was dispensed into each well. An ATBS stock solution was prepared by dissolving 22 mg of 2,2’-azino-bis (3-ethylbenzothiazoline-6-sulfonic acid) diammonium salt in 100 mL of 50 mM sodium citrate (pH 4.0). After incubating at RT for 1 h in dark conditions using an agitation machine, the plate was read at 410 nm (ELISA).

### VDBP-Complex concentration decrease test using synthesized peptide

Based on the sequences of phages that specifically bind to the VDBP-Complex, a binding affinity test of the peptide sequence was performed using Dynabeads™ M-270 Amine. The peptides, which were modified with FAM at the N-terminal of the VDBP-Complex-specific phage sequence for fluorescence labeling, were synthesized by Peptron (Korea). Modification with FAM was performed to calculate the amount of peptide bound to magnetic beads through a fluorometer. Two synthesized peptides were solved in DMSO, and diluted in PBS for activation, as described in the Dynabeads™ M-270 Amine manual^24^. The peptides and magnetic beads were conjugated as described in the Dynabeads™ M-270 Amine manual^24^. Using the peptide-conjugated beads, the binding affinity of the peptide sequence to the VDBP-Complex was confirmed. VDBP-Complex and peptide-conjugated beads were reacted by stirring at RT for 1 h using a lab tilt rotator, the supernatant was collected, and Bradford assay was performed. Fluorescence was measured by the GloMax® Explorer system (USA).

### VDBP-Complex concentration decrease test using peptides partially modified from control peptide

A modified peptide was used to confirm that it is a characteristic of a sequence that specifically binds to the VDBP-Complex. The modified peptide was partially designed using the control-specific peptide sequence (C1; YEFHPMGNPLHR) as a backbone. The binding affinity test of six peptide sequences was performed using Dynabeads™ M-270 Amine. The peptides, which were modified with FAM at the N-terminal of the VDBP-Complex-specific phage sequence for fluorescence labeling, were synthesized by Peptron (Korea). Modification with FAM was performed to calculate the amount of peptide bound to magnetic beads through a fluorometer. Each synthesized peptide was dissolved in DMSO and DW, and diluted in PBS for activation, as described in the Dynabeads™ M-270 Amine manual^24^. The peptides and magnetic beads were conjugated as described in the Dynabeads™ M-270 Amine manual^24^. The binding affinity of the peptide sequence to the VDBP-Complex was confirmed using the peptide-conjugated beads. VDBP-Complex and peptide-conjugated beads were reacted by stirring at RT for 1 h using a lab tilt rotator, the supernatant was collected, and Bradford assay was performed. Fluorescence was measured by a GloMax® Explorer system (USA).

#### Data analysis

For all experiments, each data point was obtained from three independent samples conducted simultaneously for error analysis. The results are shown as average standard deviation or correlation under several experimental conditions. The data were analyzed by using SigmaPlot (Systat Software Inc., USA). A p value < 0.01 was considered significant.

## Supplementary Information

Supplementary Information.

## Data Availability

The datasets generated and/or analyzed during the current study are available in the GenBank database (NCBI) repository under the accession numbers ON493495 (SLFTKQYDYFDT sequence; BankIt2580750 MBTL_TH_M1; https://www.ncbi.nlm.nih.gov/nuccore/ON493495), ON493496 (SFTKTSTFTWRD sequence; BankIt2580750 MBTL_TH_M3; https://www.ncbi.nlm.nih.gov/nuccore/ON493496). All data generated or analyzed during this study are included in this published article and its supplementary information files.

## Abbreviations

ABTS: 2,2′-Azino-bis(3-ethylbenzothiazoline-6-sulfonic acid) diammonium salt
Ala: Alanine
Arg: Arginine
Asn: Asparagine
Asp: Aspartate
ELISA: Enzyme-linked immunosorbent assay
FAM: 5-Carboxyfluorescein
Gln: Glutamine
Glu: Glutamate
Gly: Glycine
His: Histidine
HRP: Horseradish peroxidase
Ile: Isoleucine
IPTG: Isopropyl β-d-1-thiogalactopyranoside
Leu: Leucine
Lys: Lysine
Met: Methionine
PEG: Poly(ethylene glycol)
Phe: Phenylalanine
Pro: Proline
Ser: Serine
Thr: Threonine
Trp: Tryptophan
Tyr: Tyrosine
Val: Valine
VD: Vitamin D
VDBP: Vitamin D binding protein
VDBP-Complex: Vitamin D binding protein complex
