# Supplementary material for: Screening of novel peptides that specifically interact with vitamin D bound biocomplex proteins
Source: Sci Rep. 2023 Feb 6;13:2116. doi: 10.1038/s41598-023-28881-w (PMC9901391; doi:10.1038/s41598-023-28881-w)
Supplement: Supplementary file 1 — Supplementary Information. [file 41598_2023_28881_MOESM1_ESM.pdf]

Supplementary Information (SI)

Screening of novel peptides that specifically interact with vitamin D bound biocomplex proteins

Taehwan Kim[1], Jaewoong Lee[1], Jin-Pyo Lee[2], Bit-Na Kim[1], Yang-Hoon Kim[2†], Youn-Sik Lee[1,3‡], and Jiho Min[1, 3*]

[1]Graduate School of Semiconductor and Chemical Engineering, Jeonbuk National University, 567 Baekje-daero, Deokjin-gu, Jeonju-si, Jeollabuk-do, 54896, Republic of Korea

[2]School of Biological Science, Chungbuk National University, Chungdae-ro 1, Seowon-Gu, Cheongju, Chungbuk, 28644, Republic of Korea

[3]School of Chemical Engineering, Jeonbuk National University, 567 Baekje-daero, Deokjin-gu, Jeonju-si, Jeollabuk-do, 54896, Republic of Korea

***Corresponding Author:**
(Phone) +82-63-270-2436
(Fax) +82-63-270-2306
(E-mail) jihomin@jbnu.ac.kr

[†]Co-corresponding Author
(Phone) +82-43-261-3575
(Fax) +82-43-264-9600
(E-mail) kyh@chungbuk.ac.kr

[‡]Co-corresponding Author
(Phone) +82-63-270-2312
(Fax) +82-63-270-2306
(E-mail) yosklear@jbnu.ac.kr

Submitted to the Scientific Reports

SUPPLEMENTARY FIGURE LEGENDS

Supplementary Figure S1. Comparative analysis of the duplicate sequences appearing in the control using the pre-binding method.

There are four outstanding overlapping sequences that are observed from the negative control. The number of overlapping occurrences for each sequence is presented. (A) In the 3rd biopanning, the number of repeated occurrences of each sequence, which is observed from the control and VDBP-Complex or VDBP, is presented. (B) In the 4th biopanning, the number of repeated occurrences of each sequence, which is observed from the control and VDBP-Complex or VDBP, is presented.

Supplementary Figure S2. Overview of newly modified biopanning including pre-binding method and after-binding method.

This is an easy-to-understand illustration of how the pre-binding phage display method or after-binding phage display method works in the newly modified biopanning.

Supplementary Figure S3. Difference between traditional phage display method and changed phage display methods.

This is an easy-to-understand illustration of how the pre-binding phage display method or after-binding phage display method works.

SUPPLEMENTARY TABLE LEGENDS

Supplementary Table S1. DNA sequences analysis data from the two latest biopannings using each phage display method (horizontally expanded version)

DNA sequence analysis was performed from the 40 clones for each biopanning. In the pre-binding phage display method, underlined sequences indicate a sequence that appears simultaneously in the negative control and VDBP-Complex or VDBP. These sequences were ordered by the highest frequency appearance and significance.

(Freq : Frequency)

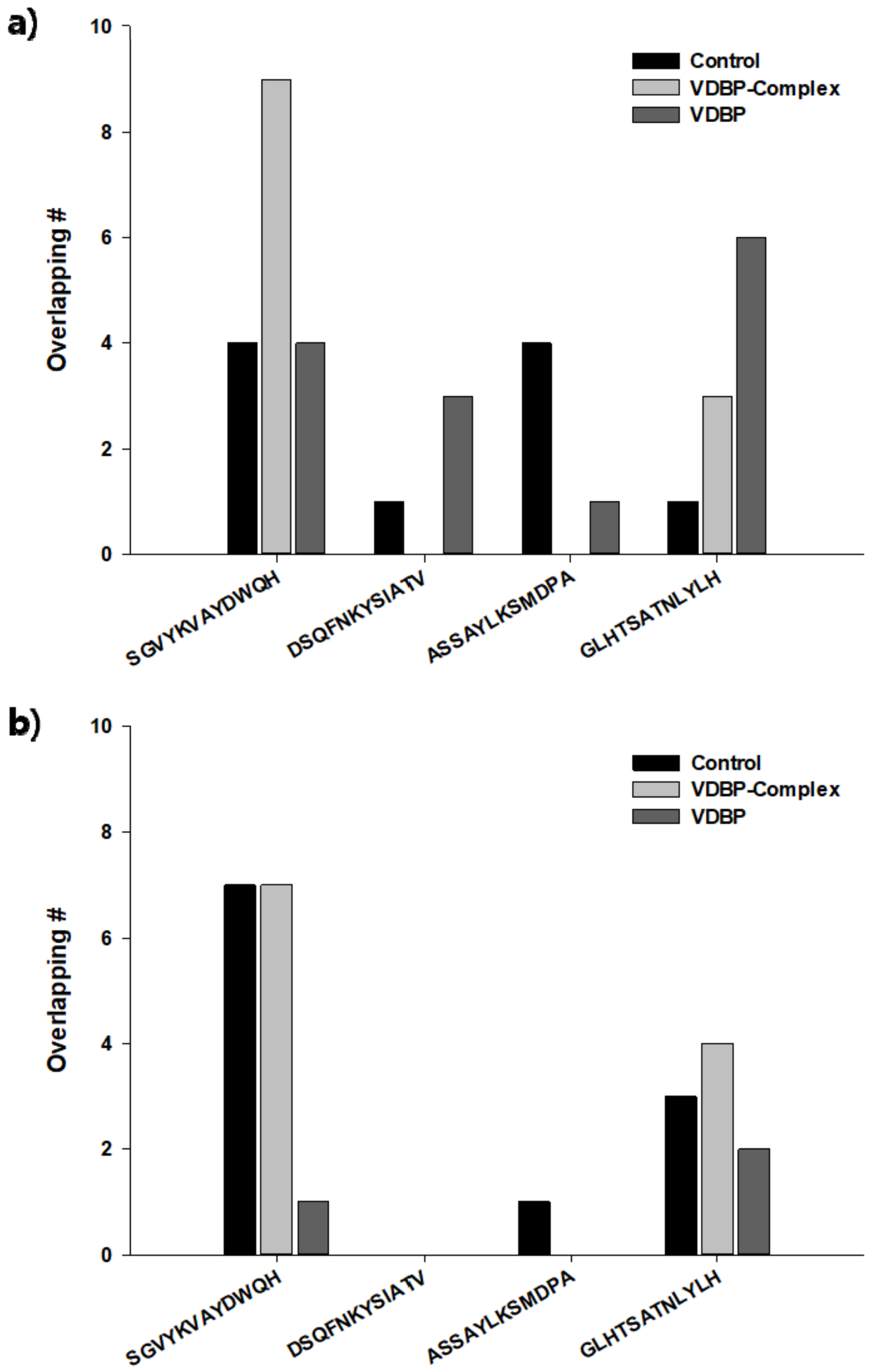

Supplementary Figure S1. Comparative analysis of the duplicate sequences appearing in the control using the pre-binding method. There are four outstanding overlapping sequences that are observed from the negative control. The number of overlapping occurrences for each sequence is presented. (a) In the 3rd biopanning, the number of repeated occurrences of each sequence, which is observed from the control and VDBP-Complex or VDBP, is presented. (b) In the 4th biopanning, the number of repeated occurrences of each sequence, which is observed from the control and VDBP-Complex or VDBP, is presented.

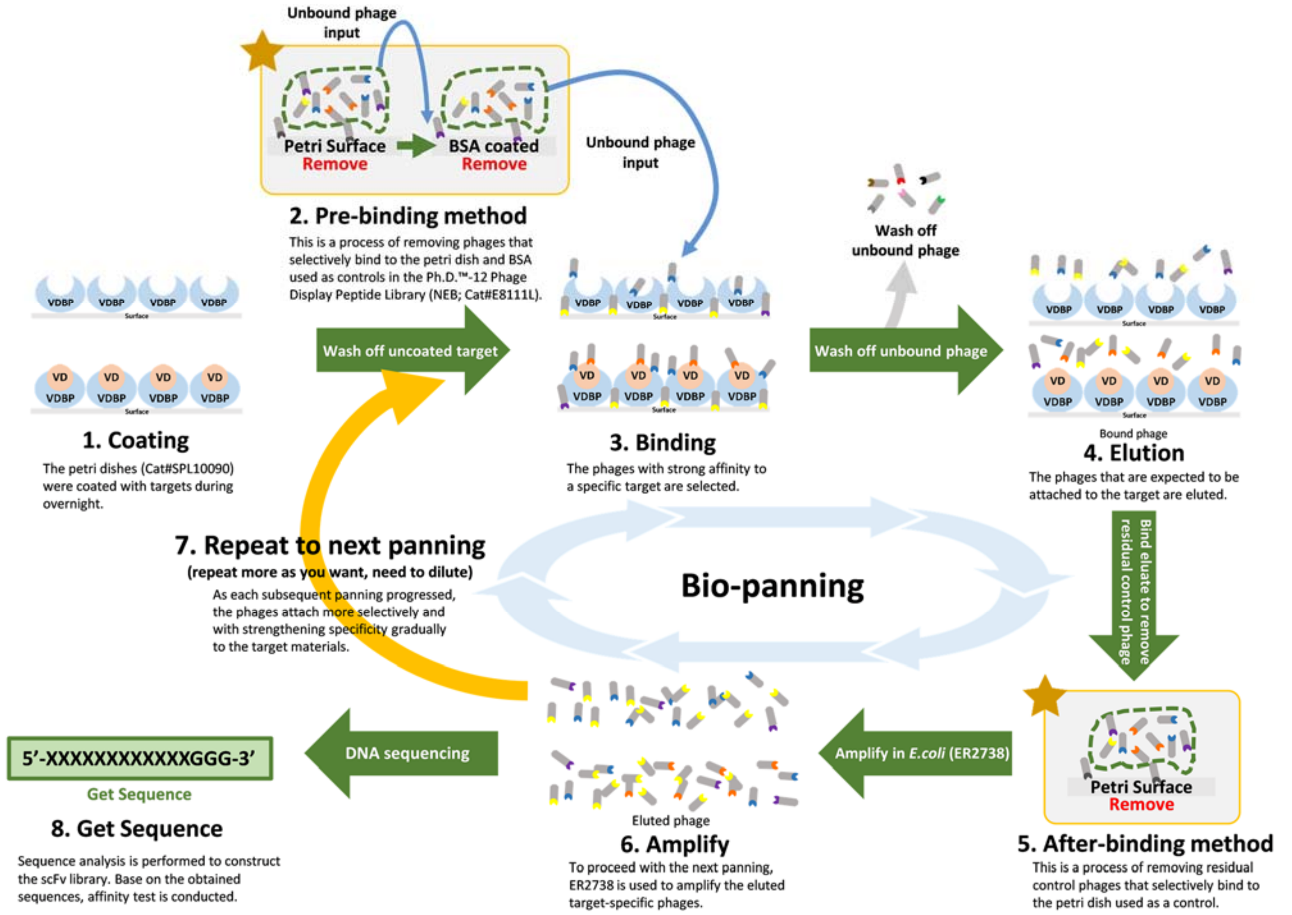

Supplementary Figure S2. Overview of newly modified biopanning including pre-binding method and after-binding method. This is an easy-to-understand illustration of how the pre-binding phage display method or after-binding phage display method works in the newly modified biopanning.

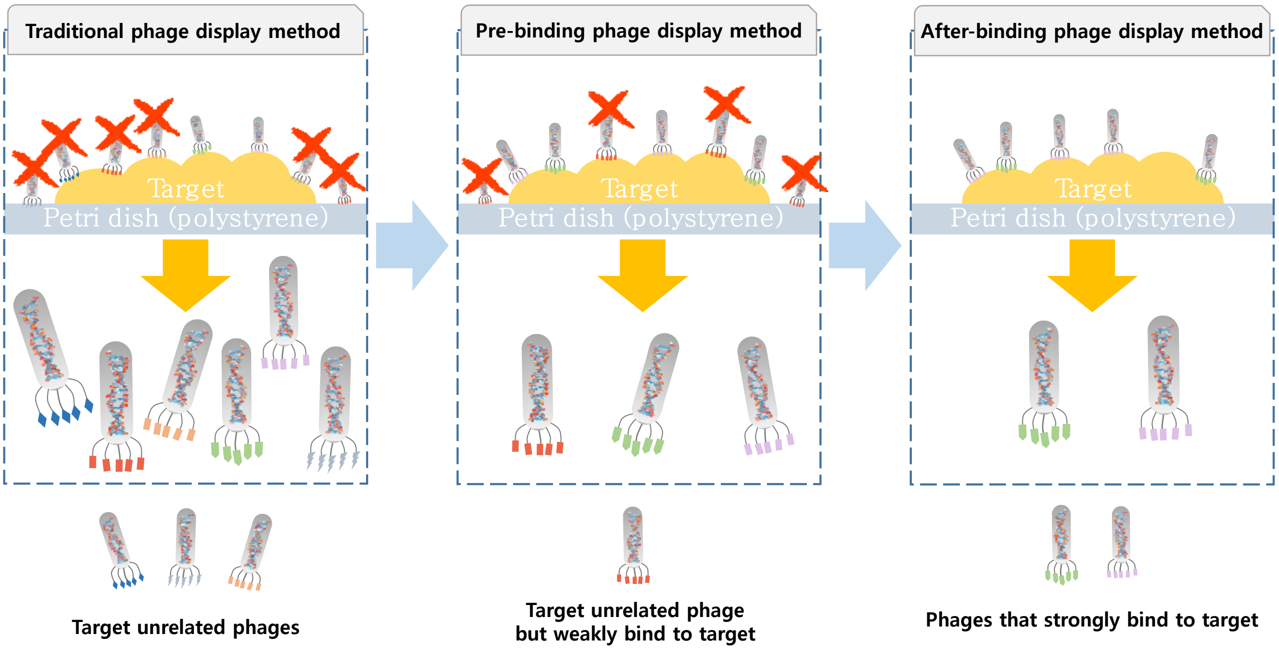

Supplementary Figure S3. Difference between traditional phage display method and changed phage display methods. This is an easy-to-understand illustration of how the pre-binding phage display method or after-binding phage display method works.

Supplementary Table S1. DNA sequences analysis data from the two latest biopannings using each phage display method (horizontally expanded version)

DNA sequence analysis was performed from the 40 clones for each biopanning. In the pre-binding phage display method, underlined sequences indicate a sequence that appears simultaneously in the negative control and VDBP-Complex or VDBP. These sequences were ordered by the highest frequency appearance and significance.

Traditional phage display method											
BSA (Negative control)				VDBP-Complex				VDBP			
2nd Panning		3rd Panning		2nd Panning		3rd Panning		2nd Panning		3rd Panning	
Sequence	Frequency	Sequence	Frequency	Sequence	Frequency	Sequence	Frequency	Sequence	Frequency	Sequence	Frequency
ETNTAGHTSLES	1	DTYSHQMKIRVP	1	AIPIWTISEVSL	1	ATFPPINSRTPA	1	APLPSDRSMNPS	1	AHASDRPSQHRV	1
FSPHNLTYNMDA	1	HHGLYRMPVTIE	1	ESWQPVHGLIPL	1	FETTYMYIKSNP	1	ATFNSQFFSKKG	1	AQPLSVYEMDPK	1
GVTDPFFDQHAE	1	HLSYDRSVLLPT	1	FEDSDAFRKFTM	1	FKTPDDSLWPHA	1	DPHWASLLDSVS	1	DDIRPQLSYHGR	1
LSPLSPPMRPLK	1	LPPHAARTPSEF	1	FHSRMLPGRLVP	1	FPLSLGSVSPLN	1	FHEIHTMPLRYA	1	HLTATELANSYH	1
LVAPLDSTAPVL	1	MHPSTSWLDSTP	1	FNSISDAGTGCT	1	GIYPFAQSSTYP	1	HHSLIPPSPVAW	1	HNSGILRTMGAY	1
…	…	…	…	…	…	…	…	…	…	…	…
WVNNSLATPYMS	1	STVGPMSTLNRS	1	SNPFALPISTQD	1	TNPLDARFHEPT	1	TNYIYRYSVDNQ	1	TSGTIFYGNSDV	1
YAPHLSTMLQYH	1	TSSAQLRHGPLL	1	TSLHGDPFHRMH	1	TNQSSQHVLIKE	1	VLAKQHSSVPLQ	1	TTSRVPDNIRLT	1
YDTPNNYFINYY	1	WPDLVHTSDSRT	1	WSTERYSATRYI	1	TSLPFPLASRHA	1	WPNAAPSGADSP	1	TYTLMNPSAMPQ	1
YSSPLMNDAKFP	1	YPVRAVPNQSGQ	1	YLDPVPKANIWL	1	YPDPLIESPKLG	1	WTPDCTLSWISS	1	YPSSVHVQWKLL	1

Pre-binding phage display method											
BSA (Negative control)				VDBP-Complex				VDBP			
3rd Panning		4th Panning		3rd Panning		4th Panning		3rd Panning		4th Panning	
Sequence	Frequency	Sequence	Frequency	Sequence	Frequency	Sequence	Frequency	Sequence	Frequency	Sequence	Frequency
ASSAYLKSMDPA	4	SGVYKVAYDWQH	7	SGVYKVAYDWQH	9	SGVYKVAYDWQH	7	GLHTSATNLYLH	6	GLHTSATNLYLH	2
SGVYKVAYDWQH	4	GLHTSATNLYLH	3	GLHTSATNLYLH	3	GLHTSATNLYLH	4	SGVYKVAYDWQH	4	SLDGAGAALRTS	2

ATDFLPYYHGLL	1	TGAPPRLDARPA	1	AFHPR*METQMY	1	GLHTPIPFVVPFYCH	1	DSQFNKYSIATV	3	DRWVARDPASIF	1
DSQFNKYSIATV	1	ASSAYLKSMDPA	1	GLHTSIPFVVPFYCH	1	SGVYTIPLVVPFYSH	1	GQSEHHMRVASF	2	GDGNSVLKPGNW	1
GDGNSVLKPGNW	1	HTAHVQADRPTQ	1	GSAPLLTVDTSK	1	SLDGAGAALRTS	1	ASSAYLKSMDPA	1	HTPMSSRLSTAS	1
GLHTSATNLYLH	1			…	…	T*TVSTENSKWW	1	…	…	…	…
GSAPLLTVDTSK	1			QWNWPVRSVANV	1			GIATMPPTFSKQ	1	SGVYKVAYDWQH	1
SGALHKSWYAGP	1			SLDGSGAALRTS	1			RTPEMTSLMAWG	1	SKGDSLPFPFAT	1
				SNVPQVPVMGHY	1			VVSPDMNLLLTN	1	SNSIDKVNRPIN	1
				SPFPGVMVHKNN	1			VVSRLPYDRVEA	1	VVSPDMNLLLTN	1

After-binding phage display method

Petri dish (Negative control)				VDBP-Complex				VDBP			
6th Panning		7th Panning		6th Panning		7th Panning		6th Panning		7th Panning	
Sequence	Frequency	Sequence	Frequency	Sequence	Frequency	Sequence	Frequency	Sequence	Frequency	Sequence	Frequency
YEFHPMGNPLHR	21	YEFHPMGNPLHR	21	SLFTKQYDYFDT	5	SLFTKQYDYFDT	6	TGSAKFLQRDTH	3	TGSAKFLQRDTH	3
SYPSNALSLHKY	1	HDPRMEHSLPKS	5	SFTKTSTFTWRD	2	VPTTSHRVAVLS	3	AFADGYSARRNL	1	ATWWQPDARGTP	1
GTGGVHPATKLT	1	NTTYPTVYADKS	1	AMPPTDLELHSK	2	FSPQNHKPNPVT	2	AFKPTSGLAKLS	1	ATYQNWTLPHRV	1
HDPRMEHSLPKS	1	SYWYEASSYTGV	1	ANGTAHSTPLLW	2	AMPPTDLELHSK	1	AWRPFPSATSGP	1	AWRPSSASTLWN	1
ISAKPIPISMRN	1	TNENLMVRLTHA	1	DRPAHGILEASL	2	IPQRYAPVSNLP	1	FAPYNNLSDNYP	1	GSAARTISPSLL	1
…	…			FSPQNHKPNPVT	1	…	…	…	…	…	…
SYPSNALSLHKY	1			IPQRYAPVSNLP	1	NNSHYYRNIFYT	1	YHGQISANAHGW	1	SPAKPHSFYTGS	1
TASSINLHAAHE	1			…	…	SSAPSMVMSTLF	1	YSSIAPSISNAL	1	SVPLNSWSIFPR	1
TLNVPPAKRSLS	1			NNSHYYRNIFYT	1	SWNHAGQPLTVV	1	YSSTSYRALTLG	1	TADVFSSSRYTR	1
TSLSTAHPMLYQ	1			TSTLYTRAQLWN	1	SYPSNALSLHKY	1	YTSLPTEATDRT	1	VQFTPRSYQPIY	1
